# Antiviral Treatment of Chronic Hepatitis B Virus (HBV) Infections^†^

**DOI:** 10.3390/v2061279

**Published:** 2010-05-31

**Authors:** Erik De Clercq, Geoffrey Férir, Suzanne Kaptein, Johan Neyts

**Affiliations:** Rega Institute for Medical Research, K.U. Leuven, Minderbroedersstraat 10, B-3000 Leuven, Belgium; E-Mails: geoffrey.ferir@rega.kuleuven.be (G.F.); suzanne.kaptein@rega.kuleuven.be (S.K.)

**Keywords:** antiviral therapy, HBV therapy, nucleoside analogs, viral hepatitis

## Abstract

While 25 compounds have been formally licensed for the treatment of HIV infection (AIDS), only seven licensed products are currently available for the treatment of chronic hepatitis B virus (HBV) infection: interferon-α, pegylated interferon-α, lamivudine, adefovir (dipivoxil), entecavir, telbivudine and tenofovir (disoproxil fumarate). In contrast to the treatment of HIV infections where the individual drugs are routinely used in combination, for the treatment of chronic HBV infection the individual drugs are generally used in monotherapy. In principle, combination drug therapy should allow reducing the likelihood of drug-resistant development.

## 1. Introduction

Chronic hepatitis B (CHB) can lead to life-threatening conditions like cirrhosis and hepatocellular carcinoma (HCC). Cirrhosis develops in approximately 20% of chronically infected patients, subsequently leading to hepatic insufficiency and portal hypertension [1]. Moreover, these patients have a 100-fold higher risk of developing hepatocellular carcinoma than non-carriers [2–4]. Hepatitis B excreted antigen (HBeAg) represents an important marker for HCC, since HBeAg-positive subjects are at highest risk of developing HCC [5]. In late stages of cirrhosis or HCC, liver transplantation is the only option left. Therefore, detection of HBV infection at an early stage and prompt treatment are of crucial importance. Indicators for a sustained virological response are clearance of HBeAg, seroconversion from HBeAg to corresponding anti-HBe antibodies, and a drop in circulating HBV DNA below detection level [1,6].

Seven drugs have been licensed by the United States FDA (Food and Drug Administration) for the treatment of CHB: interferon-alpha and pegylated interferon-alpha, three nucleoside analogs (lamivudine, entecavir and telbivudine) and two nucleotide analog prodrugs (adefovir dipivoxil and tenofovir disoproxil fumarate) [7,8]. HBV DNA polymerase is the main target for the nucleoside or nucleotide analogs. Drug combination therapy, as is now (standard care) for the treatment of HIV infections, may in the future also become adopted for the treatment of HBV infections. Various dual or triple drug combinations that have been used, with success, in the treatment of human immunodeficiency virus (HIV) infection remain to be explored for their potential in the treatment of chronic hepatitis B. Other strategies whether targeted at the viral DNA polymerase or other molecular events in the HBV replication cycle have been reported, *i.e.,* small interfering RNAs (siRNAs) {As was shown for many other viruses, siRNAs can be used to inhibit HBV replication *in vitro* [9–11] and *in vivo* [12]}, helioxanthin and related molecules, which inhibit viral nucleic acid and viral protein expression [13,14], heteroaryldihydropyrimidines (HAPs), such as BAY 41–4109 which inhibit nucleocapsid formation [15]; imino sugars (such as N-nonyl-deoxynojirimycin), which were found to suppress woodchuck hepatitis virus (WHV) replication *in vivo* (woodchucks) through inhibition of protein folding and trafficking [16]; nitazoxanide, tizoxanide and other thiazolides, which inhibit both hepatitis B virus and hepatitis C virus replication [17]; HBF-0259, a tetrahydro-tetrazolo-(1,5-a)-pyrimidine which specifically inhibits HBV surface antigen secretion [18]; β-LPA, a 2,6-diaminopurine analog containing a structure showing a mode of action that remains to be elucidated [19]; and a series of 7-deazaneplanocin A analogs for which, likewise, the mode of action remains to be elucidated [20]. Efficient inhibition of HBV replication has also been achieved by hammerhead ribozymes delivered by hepatitis delta virus [21].

## 2. HBV Replication Cycle

Three types of particles are produced during replication of HBV: (i) Dane particles (infectious virions) [22], (ii) 20 nm HBsAg (hepatitis B surface antigen) spheres and (iii) variable length HBsAg filaments [23]. The latter two particles are non-infectious [24–26]. The virions contain a 3.2 kb relaxed circular (RC) DNA with four overlapping ORFs, namely P, C, S and X. The S gene encodes for three surface proteins: Large, Medium and Small HBsAg [27]. The polymerase is translated from the P ORF. The polymerase protein consists of four domains: terminal protein, spacer, RT and RNase H [28–30]. Of the seven subdomains of RT, domain C harbors the highly conserved YMDD motif, *i.e.,* the catalytic site of the enzyme [31,32]. Infected hepatocytes also contain the hepatitis B core antigen (HBcAg). HBcAg, the excreted antigen (HBeAg), is encoded from the ORF C domain [33,34].

Following interaction with (an) unknown receptor(s), RC DNA from the virion is transported to the nucleus and converted into covalently closed circular DNA (cccDNA), while the nucleocapsids remain in the cytoplasm [35]. This DNA intermediate plays an important role in viral persistence and serves as a template for transcription by host RNA polymerase II to produce four mRNA transcripts of 3.5, 2.4, 2.1 and 0.7 kb [36]. Importantly, cccDNA may be indicative of risk for HCC development, as Wong *et al.* have shown that tumor tissues had higher levels of cccDNA compared to non-tumor tissues [37].

During core particle formation, encapsidation of the pgRNA takes place and reverse transcription starts [38]. The polymerase binds at the 5’ end of the pgRNA [39,40]. The original RNA template is degraded by RNAse H, except for a small RNA oligomer. Once (-) DNA synthesis is complete, a second translocation (the RNA primer translocation) to the 5’ end of DR2 occurs and (+) DNA synthesis starts using (-) DNA as template. Finally, circularization and extension of (variable length) (+) DNA strand terminates the DNA synthesis [39,40].

In a minority of the virions [41], (+) DNA can either be (i) converted into cccDNA [42,43] or be (ii) integrated into chromosomal DNA [44–46]. The nucleocapsids are enclosed by envelope glycoproteins after budding into the endoplasmic reticulum and Golgi apparatus [47,48]. Mature virions are released through the secretory pathway. Alternatively, nucleocapsids are recycled back to the nucleus to maintain cccDNA levels (a process referred to as recycling pathway) [35]. For a more detailed description of HBV replication and gene function, see Ganem and Schneider [49]. Of all the steps in the HBV replication cycle, the reverse transcription can be considered as the most important target for current anti-HBV chemotherapy.

## 3. Currently Approved (Licensed) Anti-HBV Drugs for the Treatment of CHB

### 3.1. Lamivudine

**Figure 1:**
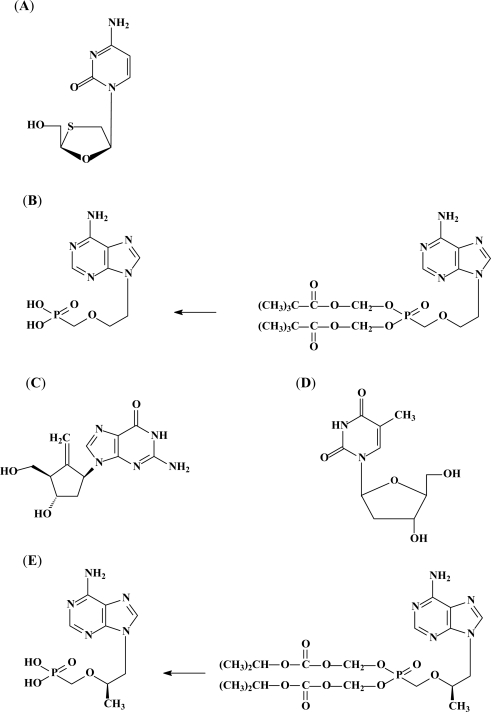
Currently licensed anti-HBV drugs. **(A)** The L-nucleoside analog lamivudine; **(B)** the acyclic nucleoside phosphonate adefovir (PMEA), licensed as its prodrug adefovir dipivoxil [bis(pivaloyloxymethyl)ester of 9-(2-phosphonylmethoxyethyl)adenine or bis(POM)PMEA]; **(C)** the carbocyclic D-nucleoside analog entecavir; **(D)** the L-nucleoside analog β-L-2’-deoxythymidine (L-dT), or telbivudine; and **(E)** the acyclic nucleoside phosphonate tenofovir, licensed as its prodrug tenofovir disoproxil fumarate (TDF) [bis(isopropoxycarbonyloxymethyl) ester of 9-(*R*)-2-(phosphonylmethoxypropyl)adenine or bis(POC)PMPA].

Lamivudine [(-)β-L-2’,3’-dideoxy-3’-thiacytidine (3TC)] (Figure 1A) has anti-HIV and anti-HBV properties. It was approved in 1998 by the FDA for the treatment of chronic hepatitis B infections. Lamivudine (Epivir-HBV^®^ or Zeffix®) is administered daily at an oral dose of 100 mg. Within the cell, 3TC is converted to its active form [3TC-triphosphate (3TC-TP)] [50]. Subsequently, 3TC-TP can act as (i) a chain terminator, after incorporation into the growing HBV DNA chain, or (ii) as a competitive inhibitor of deoxycytidine triphosphate (dCTP) at the level of the DNA polymerase. 3TC-TP inhibits viral DNA synthesis, but not mitochondrial DNA synthesis [51]. Lamivudine may interrupt the recycling of virions to the nucleus and suppress the formation of cccDNA [52].

Lamivudine treatment generally results in a three- to four-log drop in circulating HBV DNA levels, at least during the first months of treatment [53,54], and the HBV DNA levels after four weeks of lamivudine treatment may predict the long-term (five-year) outcome [55]. Concomitantly, HBeAg is cleared more rapidly from the circulation, and serum alanine aminotransferase (ALT) levels may normalize [53,54]. The virological and biochemical response may show a reduction of up to 74% and 66%, respectively [56]. The drug is usually well tolerated [57,58]. Long-term lamivudine treatment may reduce the risk of developing cirrhosis and HCC [59].

**Figure 2:**
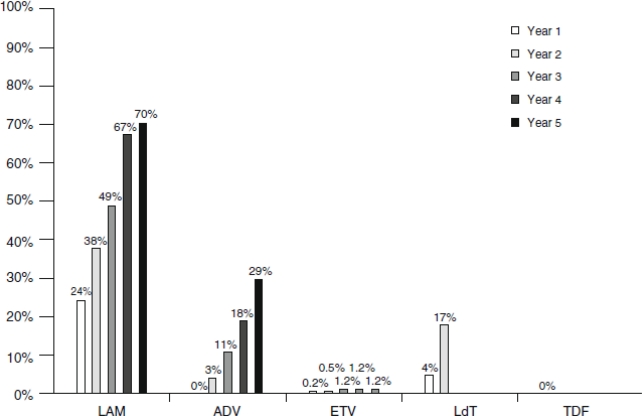
Cumulative incidence of HBV resistance to lamivudine (LAM), adefovir (ADV), entecavir (ETV), telbivudine (LdT) and tenofovir (TDF) in published pivotal trials in NUC-naive patients. For method of calculation, see [61]. These trials included different populations, used different exclusion criteria and different follow-up endpoints [151].

However, lamivudine monotherapy rapidly leads to resistance development. Approximately 20% of HBeAg-positive patients develop resistance after one year, which increases up to 70% after five years [60,61] (Figure 2). The most common mutation is observed in the catalytic YMDD motif of the viral RT polymerase. The primary lamivudine-resistance mutation is M204V/I/S (in the viral RT polymerase) in the highly conserved YMDD motif. This is often combined with the L180M mutation [62] (Table 1). Lamivudine-resistant mutations can also occur outside the YMDD motif [63]. Although lamivudine-resistant HBV mutants remain highly sensitive to adefovir and tenofovir, cross-resistance has been observed towards other L-nucleoside analogs such as emtricitabine and telbivudine [64,65].

### 3.2. Adefovir dipivoxil

Adefovir (or PMEA [9-(2-phosphonylmethoxyethyl)adenine]) is an acyclic nucleoside phosphonate [66]. To increase its oral availability, PMEA has been esterified to its prodrug bis(POM)PMEA (Figure 1B). Adefovir dipivoxil was licensed as Hepsera^®^ in September 2002 for the treatment of CHB. It is administered orally at a dose of 10 mg daily.

When PMEA enters the cell, it is phosphorylated twice by AMP kinase [67] to its active form PMEApp, which is incorporated into the growing HBV DNA chain, where it acts as (i) an obligatory chain terminator [68] and/or (ii) a competitive inhibitor of the natural substrate dATP. In addition to its anti-HBV activity, PMEApp has also demonstrated activity against other viruses, *i.e.,* herpesviruses and retroviruses as well as bacteria producing adenylate cyclase toxins (e.g., *B. anthracis*, *B. pertussis*, *P. aeruginosa*) [69]. Treatment for 48 weeks with adefovir dipivoxil led to a decrease of both cccDNA and HBsAg levels in HBeAg-positive CHB patients; it has been estimated that it may take approximately 14.5 years to clear infected cells from cccDNA [70].

Although treatment with 10 mg adefovir dipivoxil for 48 weeks resulted in a good anti-HBV response in CHB patients who were either negative or positive for HBeAg [71,72], efficacy was still improved if treatment was extended to 144 weeks in HBeAg-negative patients [73]. Adefovir dipivoxil at a dose of 10 mg/day has very few adverse effects; at higher doses, *i.e.,* 30 or 60 mg/day (or 125 mg/day as initially used when the compound was developed for HIV treatment), the compound may be nephrotoxic [1,6,72]. Adefovir dipivoxil is being used at a dose as low as 10 mg/day, which may be considered as suboptimal [74]. Adefovir dipivoxil at a dose of 10 mg is well tolerated and had a similar side effect profile as placebo in phase III clinical trials. Nephrotoxicity has been reported in 3% of patients with compensated liver disease after 4–5 years of continued adefovir dipivoxil therapy and in 6% of patients on the transplant waiting list [75,76].

The rate of development of adefovir resistance is much lower than for lamivudine monotherapy: not more than 6% after three years [73], and up to 18% after four years [77]. After five years of therapy, 29% of the treated patients harbor adefovir-resistant HBV strains (as compared to 70% for lamivudine) [78].

Recent re-assessments estimated the cumulative probability of adefovir-resistant mutations at 12 and 24 months to be 5% and 17%, respectively [79]. The main adefovir resistance is associated with the rtN236T and rtA181V/T mutations [80,81]. In lamivudine-resistant patients, emergence of the rtN236T and rtA181V/T mutations is more common (Table 1), as compared to nucleoside-naïve patients [82].

Recently, three patients with primary adefovir resistance were described, which remained sensitive to tenofovir. The HBV variant already had a mutation before adefovir therapy was even initiated. When investigated more thoroughly, results showed that an rtI233V mutation was responsible for the adefovir resistance [83]. In general, adefovir-resistant HBV mutants remain susceptible to L-nucleoside analogs (*i.e.,* lamivudine and entecavir) [84]. Adefovir resistance can be associated with viral rebound, hepatic flares and hepatic decompensation [85]. To prevent the emergence of adefovir resistance, adefovir should be combined with lamivudine, even in lamivudine-resistant patients [86]. Adefovir is active against lamivudine-resistant HBV strains (in liver transplant recipients) [76] as well as cirrhotic patients who failed on lamivudine therapy [87].

### 3.3. Entecavir

Entecavir (Figure 1C) has been approved (licensed as Baraclude^®^) in the United States for the treatment of CHB virus infections since April 2005. Its metabolism is comparable with that of the other nucleoside analogs. ETV is phosphorylated three times by human cellular kinases to its active form, ETV-TP. Intracellular accumulation occurs rapidly, its half-life is approximately 15 hours (as for lamivudine) [88] and it interferes with the HBV DNA polymerase in multiple ways: (i) it inhibits the priming of the polymerase, (ii) it has a high affinity for the HBV polymerase, (iii) it acts as a competitive inhibitor of dGTP (natural substrate) and (iv) it acts as a chain terminator two or three nucleotides downstream from its incorporation [89,90]. In fact, the incorporation of entecavir monophosphate into the DNA has at least three consequences: first, incorporation of the next nucleotide at position n + 1 following the incorporated entecavir monophosphate is compromised; second, strong pausing at position n + 3 suggests a delayed chain termination; and, third, the incorporated entecavir monophosphate can also act as a “base pair confounder” during synthesis of the second DNA strand [91].

Woodchucks chronically infected with woodchuck hepatitis virus (WHV), treated with 0.5 mg/kg ETV daily for eight weeks, showed decreased viremia levels. Long-term therapy with ETV, once a week, was also effective in maintaining low levels of viral load, decreasing cccDNA levels and viral antigens, expanding the life of the animals, and delaying the onset of HCC [92]. Two double-blind phase III studies with 715 HBeAg-positive and 648 HBeAg-negative nucleoside-naïve CHB patients revealed that entecavir therapy led to higher improved histological and virological values (like reduction in viral load, HBeAg loss and seroconversion) and reduced alanine aminotransferase levels compared with lamivudine [93,94]. Entecavir at a dose of 1 mg per day for 96 weeks of treatment resulted in continued clinical benefit in lamivudine-refractory HBeAg-positive CHB patients [95]. From clinical trials in China [96], Yao concluded that entecavir was superior to lamivudine as an anti-HBV drug [96], although in some instances, it showed only limited efficacy, with a “partial virologic response to adefovir therapy” [97].

For patients with lamivudine failure, higher doses of ETV are recommended, as 10% of these patients might develop ETV resistance after two years [89]. The approved dose of entecavir for nucleoside-naïve patients is 0.5 mg daily orally and for lamivudine-resistant patients is 1.0 mg daily orally. But even at the higher dose (1.0 mg/day), entecavir is no longer recommended for the treatment of lamivudine-resistant patients due to the high rates of entecavir resistance in such patients.

The following mutations are specifically associated with ETV resistance: rtT184G, rtS202I, and rtM250V (Table 1) [98,99]. Two additional mutations (rtM204V and rTL180M) are associated with resistance to both lamivudine and entecavir (Table 1) [99]. The virologic response is closely related to the genotypic resistance [100], and although a high incidence of the emergence of entecavir-resistant mutants has been described among patients infected with lamivudine-resistant HBV [101], long-term monitoring has shown that entecavir resistance of HBV in nucleoside-naïve patients is rare through five years of therapy [102], thus proving a high genetic barrier of HBV drug resistance to entecavir.

Surprisingly, in patients co-infected with HBV and HIV-1, entecavir led to the emergence of the lamivudine-resistant HIV-1 M184V reverse transcriptase variant [103,104], which on the one hand cautions against the use of entecavir in persons infected with both HIV-1 and HBV [105], but on the other hand suggests that entecavir may exhibit inhibitory activity against HIV under conditions of reduced viral challenge [106], although the HIV infection has no effect on the pharmacokinetics of entecavir in HBV-infected patients [107].

### 3.4. Telbivudine

Telbivudine (Figure 1D) (for recent reviews see Keam [108] and Nash [109]) offers a new option for the treatment of CHB [110]: the compound is administered orally once daily as a single tablet of 600 mg. The L-nucleoside telbivudine (β-L-2’-deoxythymidine or L-dT) is a specific anti-HBV agent. The 3’OH group is essential for anti-HBV activity; and removal or substitution of this group results in loss of activity [111]. *In vitro* studies with HepG2 cells and primary human hepatocytes have shown high phosphorylation rates of L-dT and L-dC [112]. Its active form L-dT-TP prefers to inhibit (+)-strand DNA synthesis and acts as a chain terminator [113]. It should be pointed out, however, that telbivudine, containing a 3’-hydroxyl function, does not have to act as an obligatory chain terminator.

Data from phase I and phase II clinical trials have shown that different doses of telbivudine result in considerable reductions in HBV DNA levels after four weeks of treatment. Upon withdrawal of telbivudine the viral load dramatically increased [114]. A one year trial has shown that telbivudine decreased HBV DNA levels with >6 log_10_ compared with lamivudine (∼4.5 log_10_) [115]. In HBeAg-positive, compensated CHB patients, L-dT gave an HBV DNA reduction of 6.30 log_10_ (44 patients) after 24 weeks as compared to 4.97 log_10_ for adefovir dipivoxil (89 patients) [116].

As compared to lamivudine, telbivudine demonstrated greater HBV DNA suppression in both HBeAg-negative and HBeAg-positive CHB patients [117]. Likewise, in Chinese patients with CHB, telbivudine, upon a year of treatment, provided greater antiviral and clinical efficacy than lamivudine [118]. During telbivudine treatment, non detectable serum HBV DNA after 24 weeks is the strongest predictor for optimal outcomes after two years [119].

The mutation rtM204I has been observed in patients who received telbivudine [115]. Data from Yang *et al*. showed that lamivudine-resistant strains are cross-resistant with several L-nucleosides, such as L-dT, L-dC and emtricitabine (Table 1) [64]. Therefore, telbivudine cannot be used to treat patients with lamivudine-resistant HBV. The phase III GLOBE trial showed that telbivudine, compared with lamivudine in HBeAg- positive and -negative patients, resulted in a higher antiviral and clinical efficacy after two years of treatment [120]. From a pharmacokinetic point of view, telbivudine could, in principle, be combined with lamivudine or adefovir, because no drug interactions were observed [121].

Against HBV genomes with known telbivudine-resistance mutations, M204I and L80I/M204I, telbivudine, lamivudine and entecavir lost 353- to >1000-fold activity, whereas adefovir and tenofovir exhibited no more than a 3- to 5-fold decrease in activity [122].

### 3.5. INF-α

INF-α was the first substance licensed to treat CHB virus infections. Only 30% of the patients showed a successful response with loss of HBeAg, HBV DNA and normalization of ALT levels. Influenza-like side effects were observed. The mechanism of action of interferon is two-fold [123]: (i) it elicits antiviral activity (e.g., induction of 2’,5’-oligoadenylate synthetase), as well as (ii) immunomodulatory activity (e.g., increased expression of MHC I, and stimulation of CTLs). The recommended regimen of interferon α is 5 × 106 units administered daily or 10 2 × 106 units given three times a week subcutaneously for a period of four to six months [124]. At present, two types of interferon have been approved for CHB treatment: interferon α-2b (Intron A; Schering-Plough) and pegylated interferon α-2a (PEG-IFN; Pegasys; Roche).

During 48 weeks, 814 patients with HBeAg-positive CHB received PEG-IFN plus placebo, PEG-IFN plus lamivudine, or lamivudine alone, and were followed up for 24 weeks. This study demonstrated that PEG-IFN - with or without lamivudine treatment - led to higher percentages of HBeAg seroconversion, HBV DNA suppression and HBsAg seroconversion (this was not observed with lamivudine monotherapy) and, thus, PEG-IFN 2α provides a significant improved efficiency over lamivudine [125]. In the HBeAg-negative CHB patients, PEG-IFN α-2a may offer a sustained response, resulting in HBsAg clearance three years after treatment [126].

PEG-IFN α-2b is effective against HBeAg-positive CHB, but combinations with lamivudine [127] or ribavirin [128] resulted in no additional benefit. In contrast, a recent combination treatment study in CHB patients by Wursthorn *et al*., using PEG-IFN α-2b and adefovir, showed a strong reduction in HBV DNA, cccDNA and HBsAg levels [129]. Peginterferon α-2b has been shown to be safe and effective in HBeAg-positive CHB patients with advanced fibrosis, and, hence, these patients should not be excluded from PEG-IFN treatment [130].

### 3.6. Tenofovir disoproxil fumarate

Tenofovir disoproxil fumarate (TDF) (Viread^®^) (Figure 1E) was approved by the FDA for the treatment of AIDS in 2001 and for the treatment of CHB in 2008. The efficacy of TDF against wild-type as well as lamivudine-resistant HBV strains [131] was demonstrated in patients co-infected with HIV and HBV. *In vitro* studies revealed that the combination of tenofovir with emtricitabine resulted in additive to synergistic effect. Combinations with lamivudine, entecavir or telbivudine resulted in additive effects [132]. Tenofovir undergoes two efficient phosphorylations to its active form, PMPApp, which has a long half-life (95 h) [133]. It functions as a chain terminator and represents a poor substrate for cellular DNA polymerases α, β and ɛ [134,135]. To increase oral absorption, tenofovir is esterified to its bis(isopropyloxycarbonyloxymethyl)ester [tenofovir disoproxil, bis(POC)PMPA] (Figure 1E).

At a daily dose of 300 mg it has a superior antiviral efficacy (with a similar safety profile), as compared with adefovir dipivoxil at a daily dose of 10 mg, following 48 weeks of treatment [136]. Several studies have corroborated that TDF is more potent than adefovir dipivoxil in the treatment of CHB [137–140]. TDF is highly efficacious in the treatment of advanced liver disease in patients co-infected with HBV and HIV who developed resistance to lamivudine [141]. Woodchuck studies using different concentrations of TDF administered once a day for four weeks showed a good safety profile and reductions in viremia levels [142].

The rtA194T mutation *in vitro* and in HBV/HIV co-infected patients showed TDF resistance in the presence of lamivudine mutations rtL180M and rtM204V (Table 1) [143]. The rTA194T polymerase mutations are associated with partial tenofovir resistance and negatively impacts replication competence of HBV constructs. Viral replication, however, can be restored to wild-type levels, if these polymerase mutations occur together with precore or basic core promoter substitutions as found in HBeAg-negative hepatitis B [144]. Patients with HBeAg-negative chronic HBV infection may therefore be at particular risk when developing resistance to tenofovir. TDF offers an important alternative for patients with low lamivudine or adefovir dipivoxil responses [145].

## 4. Novel anti-HBV agents

### 4.1. Emtricitabine

Emtricitabine or (-)FTC has been licensed for the treatment of HIV infections. The mechanism of action is similar to that of lamivudine. Following three phosphorylations, (-)FTC acts as (i) a chain terminator for the nascent HBV DNA chain and/or (ii) as a competitive inhibitor of its natural substrate dCTP. Additionally, (-)FTC-TP is a weak inhibitor of cellular and mitochondrial DNA polymerases. At different doses, emtricitabine, when given to chronically WHV-infected woodchucks, caused reduced viremia levels [146].

Studies in humans have shown that 200 mg (-)FTC (the optimal dose) daily for two years gave a safe antiviral profile but a resistance rate of 18% [147] (*versus* 20% for lamivudine after one year). The (-)FTC resistance mutations observed were rtM204I/V ± rtL180M and rtV173L (Table 1). A study comparing the combination of the standard dose (-)FTC and 10 mg clevudine and (-)FTC alone for 24 weeks showed no significant difference between both groups, but the combination group had a significantly greater virological and biochemical response at 24 weeks post-treatment. The prolonged anti-HBV activity of clevudine was also observed in the combination group, as well as in phase II clinical trials where clevudine monotherapy was administered [148,149]. Possible side effects observed with (-)FTC include lactic acidosis and hepatoxicity [150]. In addition, flare-ups of HBV infection after withdrawal of (-)FTC have also been reported [150].

### 4.2. Clevudine

Clevudine corresponds to L-FMAU (2’-fluoro-5-methyl-β-L-arabinofuranosyluracil). Serum HBV DNA levels were undetectable by PCR at the end of treatment in 59% of HBe Ag-positive and in 92% of HBe Ag-negative patients [151,152]. A unique feature of clevudine is the durability of viral suppression, persisting for up to 24 weeks after withdrawal of treatment. Nonetheless, clevudine has not been shown to increase the rate of HBe Ag seronconversion compared to placebo controls. Clinical trials found that the rtA181T mutation, which is associated with resistance to lamivudine and adefovir could be selected after only 24 weeks of clevudine treatment [151]. Clevudine was then reported to be associated with myopathy in patients who had been treated for longer than 24 weeks, and mitochondrial toxicity was documented in some patients [153,154]. These observations have led to the discontinuation of the global phase III clinical trials with clevudine.

## 5. Recommendations

Clinical practice guidelines for the management of chronic hepatitis B have been published by the European Association for the Study of the Liver (EASL) [155], the American Association for the Study of Liver Diseases (AASLD) [156] and the Asian Pacific Association for the Study of the Liver (APASL) [157,158]; the latter being specific for the immunomodulatory therapy of CHB.

**Figure 3:**
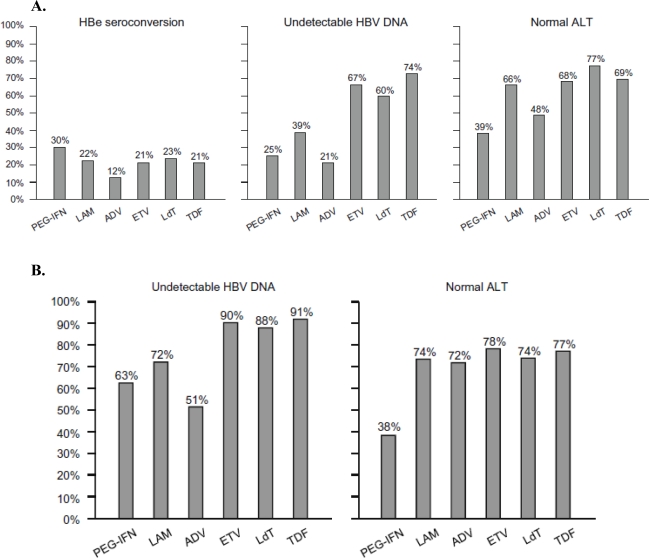
**(A)** Rates of HBe seroconversion, undetectable HBV DNA and normal ALT at one year of therapy with pegylated interferon alpha-2a (PEG-IFN), lamivudine (LAM), adefovir (ADV), entecavir (ETV), telbivudine (LdT) and tenofovir (TDF) in HBeAg-positive patients with CHB in randomized clinical trials. **(B)** Rates of undetectable HBV DNA and normal ALT at one year of therapy with PEG-IFN, LAM, ADV, ETV, LdT and TDF in HBeAg-negative patients with CHB in randomized clinical trials. The trials in **(A)** and **(B)** used different HBV DNA assays and they were not head-to-head comparisons for all the drugs; thus, these numbers are only indicative and should be considered with caution [155].

Rates of HBe seroconversion, undetectable HBV DNA and normal ALT at one year of therapy with (pegylated) interferon, lamivudine, adefovir dipivoxil, entecavir, telbivudine and tenofovir disoproxil fumarate in HBe Ag-positive patients are presented in Figure 3A, and those of undetectable HBV DNA and normal ALT in HBe Ag-negative patients are presented in Figure 3B (both sets of data taken from [155]). The most potent drugs, *i.e.,* tenofovir or entecavir, should be used as first-line monotherapies [155].

Rates of resistance at up to five years of treatment are shown in Figure 2. Resistance is associated with prior treatment with lamivudine, adefovir, telbivudine or emtricitabine. Resistance should be identified as early as possible before clinical breakthrough (increased ALT) by means of HBV DNA monitoring, and, if possible, identification of the resistance mutations. Clinical and virological studies have demonstrated the benefit of an early treatment adaptation as soon as viral load increases [159,160].

Table 2 shows cross-resistance data for the most frequent drug-resistant HBV variants [161]. Although the long-term safety of the indicated combination is unknown, it is recommended [155] to (i) in the case of lamivudine resistance, to add tenofovir (or adefovir if tenofovir is not available); (ii) in the case of adefovir resistance, to switch to tenofovir (if available; or if an N236T mutation is present, to add lamivudine, entecavir or telbivudine, or if an A181T/V mutation is present, to add entecavir); (iii) in the case of telbivudine resistance, add tenofovir (or adefovir if tenofovir is not available); (v) resistance to tenofovir has not been described so far [61,155].

The APASL [157] guidelines stipulated that for patients who develop lamivudine resistance, add-on adefovir therapy is indicated, and that for lamivudine-naïve patients who develop drug resistance while on adefovir, add-on or switching to lamivudine, telbivudine or entecavir is indicated, while for patients who develop drug resistance while on telbivudine, add-on adefovir therapy is indicated [157].

## 6. Concluding Remarks

Besides interferon, five licensed products are currently available for the treatment of chronic HBV infection: (i) lamivudine, (ii) adefovir (dipivoxil), (iii) entecavir, (iv) telbivudine and (v) tenofovir (disoproxil fumarate) (see Table 3). Lamivudine monotherapy is associated with higher resistance (up to 70% after five years) than adefovir (29% after five years) or telbivudine (9–22% after two years) [162].

Entecavir resistance is rare in naïve individuals (<1% after four years), but increases over time in lamivudine-resistant patients (43% after four years). According to Papatheodoridis *et al.* [162], the best strategy for long-term therapy in chronic HBV infection has yet to be established. This paper was published on 8 March 2008 before TDF was formally licensed for use in the treatment of CHB. Entecavir and tenofovir may represent the drugs of choice for the treatment of CHB as they couple high potency with a high genetic barrier [163], but entecavir monotherapy certainly is not the treatment of choice for lamivudine-resistant HBV patients [150].

Cross-trial comparisons of treatment outcomes, comparing pegylated interferon α-2a, lamivudine (LAM), adefovir (ADV) dipivoxil, entecavir (ETV), telbivudine (LDT) and tenofovir disoproxil fumarate (TDF) in terms of three parameters, (i) HBV DNA reduction, (ii) HBeAg seroconversion and (iii) rate of resistance have indicated that ETV and TDF (both as monotherapy) are superior to the other treatment regimens (also as monotherapy) [164]. In the near future, improvements in HBV therapy will likely involve combinations of potent nucleoside analogs (such as entecavir or telbivudine) with a potent nucleotide such as tenofovir. In the longer term future HBV therapy may or should aim at achieving a gain in post-treatment durable response rates by the development of HBV drugs with a novel mechanism of action (such as for example encapsidation inhibitors) or immunomodulatory approaches, that can be used in combination with nucleos(t)ides.

## Figures and Tables

**Table 1:** Currently licensed anti-HBV drugs as well as anti-HBV agents that are in clinical or preclinical development. Mutations that are/can be selected by these agents are indicated as well as the cross-resistance and sensitivity profiles of the drug-resistant variants.

**Agent**	**Mutation(s)**	**Cross-resistant to**	**Sensitive to**
Lamivudine	rtM204I/S/V ± rtL180M ± rtV173C rTA181S plus rTM204I rT181T	Other L-nucleoside analogs (Emtricitabine, Telbivudine) Adefovir	Adefovir, Tenofovir, Entecavir ± MPA
Adefovir	rtA181V/T and/or rtN236T		Lamivudine, Entecavir, Emtricitabine, Tenofovir
Adefovir	rtI233V		Tenofovir
Entecavir	$\begin{array}{l} \text{rtT}184\text{G}\;\text{or}\\ \text{rtS}202\text{I or}\\ \text{rtM}250\text{V}\\ \text{rtM}204\text{I} \end{array}\}\begin{array}{c} +\text{Lamivudine}-\\ \text{resistance}\\ \text{mutations} \end{array}$	Lamivudine	Adefovir
Telbivudine		Lamivudine	
Tenofovir	rtA194T + Lamivudine resistance mutations (rtM204V + rtL180M)		
Emtricitabine	rt204I/V ± rtL180M and rtV173C	Lamivudine	

**Table 2:** Cross-resistance data for the most frequently resistant HBV variants. The amino-acid substitution profiles are shown in the left column and the level of susceptibility is given for each drug: S (sensitive), I (intermediate/reduced susceptibility), R (resistant) [161].

**HBV variant**	**Level of susceptibility**				
	**Lamivudine**	**Telbivudine**	**Entecavir**	**Adefovir**	**Tenofovir**
Wild-type	S	S	S	S	S
M204I	R	R	I	S	S
L180M + M204V	R	R	I	S	S
A181T/V	I	S	S	R	S
N236T	S	S	S	R	I
L180M + M204I/S/V ± I169T ± V173L ± M250V	R	R	R	S	S
L180M + M204I/S/V ± T184G ± S202I/G	R	R	R	S	S

**Table 3:** Anti-HBV agents.

**Generic name**	**Brand name**	**Manufacturer**	**Date of FDA approval**
Anti-HBV drugs approved by the US Food and Drug Administration			
Interferon alfa-2b	Intron A	Schering-Plough	13 July 1992
Lamivudine, 3TC	Epivir-HBV	GlaxoSmithKline	9 December 1998
Adefovir dipivoxil	Hepsera	Gilead Sciences	20 September 2002
Entecavir	Baraclude	Bristol-Myers Squibb	30 March 2005
Peginterferon alfa-2a	Pegasys	Roche	13 May 2005
Telbivudine	Tyzeka	Idenix	25 October 2006
Tenofovir disoproxil fumarate	Viread	Gilead Sciences	11 August 2008

## Abbreviations

3TC: Lamivudine [(-)β-L-2’,3’-dideoxy-3’-thiacytidine]
3TC-TP: 3TC-triphosphate
cccDNA: covalently closed circular DNA
CHB: Chronic Hepatitis B
dCTP: Deoxycytidine Triphosphate
DDDP: Viral DNA - Dependent DNA Polymerase
DHBV: Duck Hepatitis B Virus
DR: Direct Repeat
dsl: double-stranded linear
EC_50_: 50% Effective (inhibitory) Concentration
FDA: Food and Drug Administration
(-)FTC: Emtricitabine
HBcAg: Hepatitis B core Antigen
HBeAg: Hepatitis B e Antigen
HBsAg: Hepatitis B surface Antigen
HBV: (Human) Hepatitis B Virus
HCC: Hepatocellular Carcinoma
L-dT: β-L-2′-deoxythymidine
NNRTIs: Non-Nucleoside Reverse Transcriptase Inhibitors
PEG-IFN: Pegylated Interferon α-2a
pgRNA: pregenomic RNA
RC: Relaxed Circular
siRNAs: small interfering RNAs
TDF: Tenofovir Disoproxil Fumarate
WHV: Woodchuck Hepatitis Virus

## References

[1] Ganem D, Prince AM (2004). Hepatitis B virus infection - Natural history and clinical consequences. N Engl J Med.

[2] Beasley RP (1988). Hepatitis B virus: the major etiology of hepatocellular carcinoma. Cancer.

[3] Chiaramonte M, Stroffolini T, Vian A, Stazi MA, Floreani A, Lorenzoni U, Lobello S, Farinati F, Naccarato R (1999). Rate of incidence of hepatocellular carcinoma in patients with compensated viral cirrhosis. Cancer.

[4] Fattovich G, Pantalena M, Zagni I, Realdi G, Schalm SW, Christensen E (2002). Effect of hepatitis B and C virus infections on the natural history of compensated cirrhosis: a cohort study of 297 patients. Am J Gastroenterol.

[5] Yang HI, Lu SN, Liaw YF, You SL, Sun CA, Wang LY, Hsiao CK, Chen PJ, Chen DS, Chen CJ (2002). Hepatitis B e antigen and the risk of hepatocellular carcinoma. N Engl J Med.

[6] Zoulim F (2006). Antiviral therapy of chronic hepatitis B. Antiviral Res.

[7] Keeffe EB, Marcellin P (2007). New and emerging treatment of chronic hepatitis B. Clin Gastroenterol Hepatol.

[8] Palumbo E (2008). New drugs for chronic hepatitis B: a review. Am J Therapeut.

[9] Peng J, Zhao Y, Mai J, Pang WK, Wei X, Zhang P, Xu Y (2005). Inhibition of hepatitis B virus replication by various RNAi constructs and their pharmacodynamic properties. J Gen Virol.

[10] Ying C, De Clercq E, Neyts J (2003). Selective inhibition of hepatitis B virus replication by RNA interference. Biochem Biophys Res Commun.

[11] Chen Y, Cheng G, Mahato RI (2008). RNAi for treating hepatitis B viral infection. Pharm Res.

[12] McCaffrey AP, Nakai H, Pandey K, Huang Z, Salazar FH, Xu H, Wieland SF, Marion PI, Kay MA (2003). Inhibition of hepatitis B virus in mice by RNA interference. Nat Biotechnol.

[13] Cheng YC, Ying CX, Leung CH, Li Y (2005). New targets and inhibitors of HBV replication to combat drug resistance. J Clin Virol.

[14] Ying C, Li Y, Leung CH, Robek MD, Cheng YC (2007). Unique antiviral mechanism discovered in anti-hepatitis B virus research with a natural product analogue. Proc Natl Acad Sci USA.

[15] Deres K, Schröder CH, Paessens A, Goldmann S, Hacker HJ, Weber O, Krämer T, Niewöhner U, Pleiss U, Stoltefuss J, Graef E, Koletzki D, Masantschek RN, Reimann A, Jaeger R, Gross R, Beckermann B, Schlemmer KH, Haebich D, Rübsamen-Waigmann H (2003). Inhibition of hepatitis B virus replication by drug-induced depletion of nucleocapsids. Science.

[16] Block TM, Lu X, Mehta AS, Blumberg BS, Tennant B, Ebling M, Korba B, Lansky DM, Jacob GS, Dwek RA (1998). Treatment of chronic hepadnavirus infection in a woodchuck animal model with an inhibitor of protein folding and trafficking. Nat Med.

[17] Korba BE, Montero AB, Farrar K, Gaye K, Mukerjee S, Ayers MS, Rossignol JF (2008). Nitazoxanide, tizoxanide and other thiazolides are potent inhibitors of hepatitis B virus and hepatitis C virus replication. Antiviral Res.

[18] Dougherty AM, Guo H, Westby G, Liu Y, Simsek E, Guo JT, Mehta A, Norton P, Gu B, Block T, Cuconati A (2007). A substituted tetrahydro-tetrazolo-pyrimidine is a specific and novel inhibitor of hepatitis B virus surface antigen secretion. Antimicrob Agents Chemother.

[19] He XX, Chen T, Lin JS, Chang Y, Ye BX (2008). Inhibition of the replication of hepatitis B virus *in vitro* by a novel 2,6-diaminopurine analog, beta-LPA. Biochem Biophys Res Commun.

[20] Kim HJ, Sharon A, Bal C, Wang J, Allu M, Huang Z, Murray MG, Bassit L, Schinazi RF, Korba B, Chu CK (2009). Synthesis and anti-hepatitis B virus and anti-hepatitis C virus activities of 7-deazaneplanocin A analogues *in vitro*. J Med Chem.

[21] Li X, Kuang E, Dai W, Zhou B, Yang F (2005). Efficient inhibition of hepatitis B virus replication by hammerhead ribozymes delivered by hepatitis delta virus. Virus Res.

[22] Dane DS, Cameron CH, Briggs M (1970). Virus-like particles in serum of patients with Australia-antigen-associated hepatitis. Lancet.

[23] Robinson WS, Lutwick LI (1976). The virus of hepatitis, type B (first of two parts). N Engl J Med.

[24] Gavilanes F, Gonzalez-Ros JM, Peterson DL (1982). Structure of hepatitis B surface antigen: Characterization of the lipid components and their association with the viral proteins. J Biol Chem.

[25] Peterson DL (1981). Isolation and characterization of the major protein and glycoprotein of hepatitis B surface antigen. J Biol Chem.

[26] Peterson DL (1987). The structure of hepatitis B surface antigen and its antigenic sites. Bioessays.

[27] Valenzuela P, Gray P, Quiroga M, Zaldivar J, Goodman HM, Rutter WJ (1979). Nucleotide sequence of the gene coding for the major protein of hepatitis B virus surface antigen. Nature.

[28] Bartenschlager R, Schaller H (1988). The amino-terminal domain of the hepadnaviral P-gene encodes the terminal protein (genome linked protein) believed to prime reverse transcription. EMBO J.

[29] Chang LJ, Hirsch RC, Ganem D, Varmus HE (1990). Effects of insertional and point mutations on the functions of the duck hepatitis B virus polymerase. J Virol.

[30] Radziwill G, Tucker W, Schaller H (1990). Mutational analysis of the hepatitis B virus P gene product: domain structure and RNase H activity. J Virol.

[31] Poch O, Sauvaget I, Delarue M, Tordo N (1989). Identification of four conserved motifs among the RNA-dependent polymerase encoding elements. EMBO J.

[32] Tipples GA, Ma MM, Fischer KP, Bain VG, Kneteman NM, Tyrrell DLJ (1996). Mutation in HBV RNA-dependent DNA polymerase confers resistant to lamivudine *in vivo*. Hepatology.

[33] Ou JH, Laub O, Rutter WJ (1986). Hepatitis B virus gene function: the pre-core region targets the core antigen to cellular membranes and causes the secretion of the e entigen. Proc Natl Acad Sci USA.

[34] Pasek M, Goto T, Gilbert W, Zink B, Schaller H, MacKay P, Leadbetter G, Murray K (1979). Hepatitis B virus genes and their expression in E. coli. Nature.

[35] Tuttleman JS, Pourcel C, Summers J (1986). Formation of the pool of covalently closed circular viral DNA in hepadnavirus-infected cells. Cell.

[36] Will H, Reiser W, Weimer T, Pfaff E, Büscher M, Sprengel R, Cattaneo R, Schaller H (1987). Replication strategy of human hepatitis B virus. J Virol.

[37] Wong DK, Yuen MF, Poon RT, Yuen JC, Fung J, Lai CL (2006). Quantification of hepatitis B virus covalently closed circular DNA in patients with hepatocellular carcinoma. J Hepatol.

[38] Pollack JR, Ganem D (1994). Site-specific RNA binding by a hepatitis B virus reverse transcriptase initiates two distinct reactions: RNA packaging and DNA synthesis. J Virol.

[39] Hu J, Seeger C (1996). Hsp90 is required for the activity of a hepatitis B virus reverse transcriptase. Proc Natl Acad Sci USA.

[40] Seeger C, Mason WS (2000). Hepatitis B virus biology. Microbiol Mol Biol Rev.

[41] Staprans S, Loeb DD, Ganem D (1991). Mutations affecting hepadnavirus plus-strand DNA synthesis dissociate primer cleavage from translocation and reveal the origin of linear viral DNA. J Virol.

[42] Yang W, Mason WS, Summers J (1996). Covalently closed circular viral DNA formed from two types of linear DNA in woodchuck hepatitis virus-infected liver. J Virol.

[43] Yang W, Summers J (1995). Illegitimate replication of linear hepadnavirus DNA through nonhomologous recombination. J Virol.

[44] Yang W, Summers J (1999). Integration of hepadnavirus DNA in infected liver: evidence for a linear precursor. J Virol.

[45] Gong SS, Jensen AD, Chang CJ, Rogler CE (1999). Double-stranded linear duck hepatitis B virus (DHBV) stably integrates at a higher frequency than wild-type DHBV in LMH chicken hepatoma cells. J Virol.

[46] Gong SS, Jensen AD, Wang H, Rogler CE (1995). Duck hepatitis B virus integrations in LMH chicken hepatoma cells: identification and characterization of new episomally derived integrations. J Virol.

[47] Kamimura T, Yoshikawa A, Ichida F, Sasaki H (1981). Electron microscopic studies of Dane particles in hepatocytes with special reference to intracellular development of Dane particles and their relation with HBeAg in serum. Hepatology.

[48] Roingeard P, Lu SL, Sureau C, Freschlin M, Arbeille B, Essex M, Romet-Lemonne JL (1990). Immunocytochemical and electron microscopic study of hepatitis B virus antigen and complete particle production in hepatitis B virus DNA transfected HepG2 cells. Hepatology.

[49] Ganem D, Schneider RJ (2001). Hepadnaviridae: The viruses and their replication. Fields Virology.

[50] Cammack N, Rouse P, Marr CL, Reid PJ, Boehme RE, Coates JA, Penn CR, Cameron JM (1992). Cellular metabolism of (-) enantiomeric 2′-deoxy-3′-thiacytidine. Biochem Pharmacol.

[51] Chang CN, Skalski V, Zhou JH, Cheng YC (1992). Biochemical pharmacology of (+)- and (-)-2′,3′-dideoxy-3′-thiacytidine as anti-hepatitis B virus agents. J Biol Chem.

[52] Lai CL, Yuen MF (2000). Profound suppression of hepatitis B virus replication with lamivudine. J Med Virol.

[53] Dienstag JL, Schiff ER, Wright TL, Perrillo RP, Hann HW, Goodman Z, Crowther L, Condreay LD, Woessner M, Rubin M, Brown NA (1999). Lamivudine as initial treatment for chronic hepatitis B in the United States. N Engl J Med.

[54] Lai CL, Chine RW, Leung NWY, Chang TT, Guan R, Tai DI, Ng KY, Wu PC, Dent JC, Barber J, Stephenson SL, Gray F (1998). A one year trial of lamivudine for chronic hepatitis B. N Engl J Med.

[55] Yuen MF, Fong DY, Wong DK, Yuen JC, Fung J, Lai CL (2007). Hepatitis B virus DNA levels at week 4 of lamivudine treatment predict the 5-year ideal response. Hepatology.

[56] Fung SK, Wong F, Hussain M, Lok ASF (2004). Sustained response after a 2-year course of lamivudine treatment of hepatitis B e antigen-negative chronic hepatitis B. J Viral Hepatol.

[57] Perry CM, Faulds D (1997). Lamivudine. A review of its antiviral activity, pharmacokinetic properties and therapeutic efficacy in the management of HIV infection. Drugs.

[58] Schalm SW, Heathcote J, Cianciara J, Farrell G, Sherman M, Willems B, Dhillon A, Moorat A, Barber J, Gray DF (2000). Lamivudine and alpha interferon combination treatment of patients with chronic hepatitis B infection: a randomised trial. Gut.

[59] Yuen MF, Seto WK, Chow DH, Tsui K, Wong DK, Ngai VW, Wong BC, Fung J, Yuen JC, Lai CL (2007). Long-term lamivudine therapy reduces the risk of long-term complications of chronic hepatitis B infection even in patients without advanced disease. Antiviral Ther.

[60] Lok AS, Lai CL, Leung N, Yao GB, Cui ZY, Schiff ER, Dienstag JL, Heathcote EJ, Little NR, Griffiths DA, Gardner SD, Castiglia M (2003). Long-term safety of lamivudine treatment in patients with chronic hepatitis B. Gastroenterology.

[61] Pawlotsky JM, Dusheiko G, Hatzakis A, Lau D, Lau G, Liang TJ, Locarnini S, Martin P, Richman DD, Zoulim F (2008). Virologic monitoring of hepatitis B virus therapy in clinical trials and practice: recommendations for a standardized approach. Gastroenterology.

[62] Das K, Xiong X, Yang H, Westland CE, Gibbs CS, Sarafianos SG, Arnold E (2001). Molecular modeling and biochemical characterization reveal the mechanism of hepatitis B virus polymerase resistance to lamivudine (3TC) and emtricitabine (FTC). J Virol.

[63] Yatsuji H, Noguchi C, Hiraga N, Mori N, Tsuge M, Imamura M, Takahashi S, Iwao E, Fujimoto Y, Ochi H, Abe H, Maekawa T, Tateno C, Yoshizato K, Suzuki F, Kumada H, Chayama K (2006). Emergence of a novel lamivudine-resistant hepatitis B virus variant with a substitution outside the YMDD motif. Antimicrob Agents Chemother.

[64] Yang H, Qi X, Sabogal A, Miller M, Xiong S, Delaney WE (2005). Cross-resistance testing of next-generation nucleoside and nucleotide analogues against lamivudine-resistant HBV. Antiviral Ther.

[65] Karatayli E, Karayalçin S, Karaaslan H, Kayhan H, Türkyilmaz AR, Sahin F, Yurdaydin C, Bozdayi AM (2007). A novel mutation pattern emerging during lamivudine treatment shows cross-resistance to adefovir dipivoxil treatment. Antiviral Ther.

[66] De Clercq E, Holý A, Rosenberg I, Sakuma T, Balzarini J, Maudgal PC (1986). A novel selective broad-spectrum anti-DNA virus agent. Nature.

[67] Merta A, Votruba I, Jindrich J, Holý A, Cihlár T, Rosenberg I, Otmar M, Herve TY (1992). Phosphorylation of 9-(2-phosphonomethoxyethyl)adenine and 9-(S)-(3-hydroxy-2-phosphonomethoxypropyl)adenine by AMP(dAMP) kinase from L1210 cells. Biochem Pharmacol.

[68] De Clercq E (2004). Antivirals and antiviral strategies. Nat Rev Microbiol.

[69] Shen Y, Zhukovskaya NL, Zimmer MI, Soelaiman S, Bergson P, Wang CR, Gibbs CS, Tang WJ (2004). Selective inhibition of anthrax edema factor by adefovir, a drug for chronic hepatitis B virus infection. Proc Natl Acad Sci U S A.

[70] Werle-Lapostolle B, Bowden S, Locarnini S, Wursthorn K, Petersen J, Lau G, Trepo C, Marcellin P, Goodman Z, Delaney WE, Xiong S, Brosgart CL, Chen SS, Gibbs CS, Zoulim F (2004). Persistence of cccDNA during the natural history of chronic hepatitis B and decline during adefovir dipivoxil therapy. Gastroenterology.

[71] Hadziyannis SJ, Tassopoulos NC, Heathcote EJ, Chang TT, Kitis G, Rizzetto M, Marcellin P, Lim SG, Goodman Z, Wulfsohn MS, Xiong S, Fry J, Brosgart CL (2003). Adefovir dipivoxil for the treatment of hepatitis B e antigen-negative chronic hepatitis B. N Engl J Med.

[72] Marcellin P, Chang TT, Lim SG, Tong MJ, Sievert W, Shiffman ML, Jeffers L, Goodman Z, Wulfsohn MS, Xiong S, Fry J, Brosgart CL (2003). Adefovir dipivoxil 437 study group. Adefovir dipivoxil for the treatment of hepatitis B e antigen-positive chronic hepatitis B. N Engl J Med.

[73] Hadziyannis SJ, Tassopoulos NC, Heathcote EJ, Chang TT, Kitis G, Rizzetto M, Marcellin P, Lim SG, Goodman Z, Ma J, Arterburn S, Xiong S, Currie G, Brosgart CL (2005). Long-term therapy with adefovir dipivoxil for HBeAg-negative chronic hepatitis B. N Engl J Med.

[74] Carrouée-Durantel S, Durantel D, Werle-Lapostolle B, Pichoud C, Naesens L, Neyts J, Trépo C, Zoulim F (2008). Suboptimal response to adefovir dipivoxil therapy for chronic hepatitis B in nucleoside-naive patients is not due to pre-existing drug-resistant mutants. Antiviral Ther.

[75] Hadziyannis SJ, Tassopoulos NC, Heathcote EJ, Chang TT, Kitis G, Rizzetto M, Marcellin P, Lim SG, Goodman Z, Ma J, Brosgart CL, Borroto-Esoda K, Arterburn S, Chuck SL, Adefovir Dipivoxil 438 Study Group (2006). Long-term therapy with adefovir dipivoxil for HBeAg-negative chronic hepatitis B for up to 5 years. Gastroenterology.

[76] Schiff E, Lai CL, Hadziyannis S, Neuhaus P, Terrault N, Colombo M, Tillmann H, Samuel D, Zeuzem S, Villeneuve JP, Arterburn S, Borroto-Esoda K, Brosgart C, Chuck S (2007). Adefovir dipivoxil for wait-listed and post-liver transplantation patients with lamivudine-resistant hepatitis B: final long-term results. Liver Transpl.

[77] Marcellin P, Asselah T (2005). Resistance to adefovir: a new challenge in the treatment of chronic hepatitis B. J Hepatol.

[78] Borroto-Esoda K, Miller MD, Arterburn S (2007). Pooled analysis of amino acid changes in the HBV polymerase in patients from four major adefovir dipivoxil clinical trials. J Hepatol.

[79] Gallego A, Sheldon J, García-Samaniego J, Margall N, Romero M, Hornillos P, Soriano V, Enrĺquez J (2008). Evaluation of initial virological response to adefovir and development of adefovir-resistant mutations in patients with chronic hepatitis B. J Viral Hepat.

[80] Lacombe K, Ollivet A, Gozlan J, Durantel S, Tran N, Girard PM, Zoulim F (2006). A novel hepatitis B virus mutation with resistance to adefovir but not to tenofovir in an HIV-hepatitis B virus-co-infected patient. AIDS.

[81] Osiowy C, Villeneuve JP, Heathcote EJ, Giles E, Borlang J (2006). Detection of rtN236T and rtA181V/T mutations associated with resistance to adefovir dipivoxil in samples from patients with chronic hepatitis B virus infection by the INNO-LiPA HBV DR Line Probe Assay (version 2). J Clin Microbiol.

[82] Lee YS, Suh DJ, Lim YS, Jung SW, Kim KM, Lee HC, Chung YH, Lee YS, Yoo W, Kim SO (2006). Increased risk of adefovir resistance in patients with lamivudine-resistant chronic hepatitis B after 48 weeks of adefovir dipivoxil monotherapy. Hepatology.

[83] Schildgen O, Sirma H, Funk A, Olotu C, Wend UC, Hartmann H, Helm M, Rockstroh JK, Willems WR, Will H, Gerlich WH (2006). Variant of hepatitis B virus with primary resistance to adefovir. N Engl J Med.

[84] Villeneuve JP, Durantel D, Durantel S, Westland C, Xiong S, Brosgart CL, Gibbs CS, Parvaz P, Werle B, Trépo C, Zoulim F (2003). Selection of a hepatitis B virus strain resistant to adefovir in a liver transplantation patient. J Hepatol.

[85] Fung SK, Andreone P, Han SH, Reddy KR, Regev A, Keeffe EB, Hussain M, Cursaro C, Richtmyer P, Marrero JA, Lok ASF (2005). Adefovir-resistant hepatitis B can be associated with viral rebound and hepatic decompensation. J Hepatol.

[86] Fung SK, Chae HB, Fontana RJ, Conjeevaral H, Marrero J, Oberhelman K, Hussain M, Lok ASF (2006). Virologic response and resistance to adefovir in patients with chronic hepatitis B. J Hepatol.

[87] Zoulim F, Parvaz P, Marcellin P, Zarski JP, Beaugrand M, Benhamou Y, Bailly F, Maynard M, Trepo C, Trylesinski A, Monchecourt F (2009). Adefovir dipivoxil is effective for the treatment of cirrhotic patients with lamivudine failure. Liver Int.

[88] Yamanaka G, Wilson T, Innaimo S, Bisacchi GS, Egli P, Rinehart JK, Zahler R, Colonno RJ (1999). Metabolic studies on BMS-200475, a new antiviral compound active against hepatitis B virus. Antimicrob Agents Chemother.

[89] Zoulim F (2006). Entecavir: a new treatment option for chronic hepatitis B. J Clin Virol.

[90] Seifer M, Hamatake RK, Colonno RJ, Standring DN (1998). *In vitro* inhibition of hepadnavirus polymerases by the triphosphates of BMS-200475 and lobucavir. Antimicrob Agents Chemother.

[91] Tchesnokov EP, Obikhod A, Schinazi RF, Götte M (2008). Delayed chain termination protects the anti-hepatitis B virus drug entecavir from excision by HIV-1 reverse transcriptase. J Biol Chem.

[92] Colonno RJ, Genovesi EV, Medina I, Lamb L, Durham SK, Huang ML, Corey L, Littlejohn M, Locarnini S, Tennant BC, Rose B, Clark JM (2001). Long-term entecavir treatment results in sustained antiviral efficacy and prolonged life span in the woodchuck model of chronic hepatitis infection. J Infect Dis.

[93] Chang TT, Gish RG, de Man R, Gadano A, Sollano J, Chao YC, Lok AS, Han KH, Goodman Z, Zhu J, Cross A, DeHertogh D, Wilber R, Colonno R, Apelian D (2006). A comparison of entecavir and lamivudine for HBeAg-positive chronic hepatitis B. N Engl J Med.

[94] Lai CL, Shouval D, Lok AS, Chang TT, Cheinquer H, Goodman Z, DeHertogh D, Wilber R, Zink RC, Cross A, Colonno R, Fernandes L (2006). Entecavir *versus* lamivudine for patients with HBeAg-negative chronic hepatitis B. N Engl J Med.

[95] Sherman M, Yurdaydin C, Simsek H, Silva M, Liaw YF, Rustgi VK, Sette H, Tsai N, Tenney DJ, Vaughan J, Kreter B, Hindes R (2008). Entecavir therapy for lamivudine-refractory chronic hepatitis B: improved virologic, biochemical, and serology outcomes through 96 weeks. Hepatology.

[96] Yao G (2007). Entecavir is a potent anti-HBV drug superior to lamivudine: experience from clinical trials in China. J Antimicrob Chemother.

[97] Reijnders JGP, Pas SD, Schutten M, de Man RA, Janssen HLA (2009). Entecavir shows limited efficacy in HBeAg-positive hepatitis B patients with a partial virologic response to adefovir therapy. J Hepatol.

[98] Tenney DJ, Levine SM, Rose RE, Walsh AW, Weinheimer SP, Discotto L, Plym M, Pokornowski K, Yu CF, Angus P, Ayres A, Bartholomeusz A, Sievert W, Thompson G, Warner N, Locarnini S, Colonno RJ (2004). Clinical emergence of entecavir-resistant hepatitis B virus requires additional substitutions in virus already resistant to lamivudine. Antimicrob Agents Chemother.

[99] Baldick CJ, Tenney DJ, Mazzucco CE, Eggers BJ, Rose RE, Pokornowski KA, Yu CF, Colonno RJ (2008). Comprehensive evaluation of hepatitis B virus reverse transcriptase substitutions associated with entecavir resistance. Hepatology.

[100] Baldick CJ, Eggers BJ, Fang J, Levine SM, Pokornowski KA, Rose RE, Yu C-F, Tenney DJ, Colonno RJ (2008). Hepatitis B virus quasispecies susceptibility to entecavir confirms the relationship between genotypic resistance and patient virologic response. J Hepatol.

[101] Nagasaki F, Niitsuma H, Ueno Y, Inoue J, Kogure T, Fukushima K, Shimosegawa T (2007). The high incidence of the emergence of entecavir-resistant mutants among patients infected with lamivudine-resistant hepatitis B virus. Tohoku J Exp Med.

[102] Tenney DJ, Rose RE, Baldick CJ, Pokornowski KA, Eggers BJ, Fang J, Wichroski MJ, Xu D, Yang J, Wilber RB, Colonno RJ (2009). Long-term monitoring shows hepatitis B virus resistance to entecavir in nucleoside-naïve patients is rare through 5 years of therapy. Hepatology.

[103] McMahon MA, Jilek BL, Brennan TP, Shen L, Zhou Y, Wind-Rotolo M, Xing S, Bhat S, Hale B, Hegarty R, Chong CR, Liu JO, Siliciano RF, Thio CL (2007). The HBV drug entecavir - effects on HIV-1 replication and resistance. N Engl J Med.

[104] Jain MK, Zoellner CL (2007). Entecavir can select for M184V of HIV-1: a case of an HIV/hepatitis B (HBV) naïve patient treated for chronic HBV. AIDS.

[105] Sasadeusz J (2007). The anti-HIV antiviral activity of entecavir: the loss of a trusted friend. J Hepatol.

[106] Lin PF, Nowicka-Sans B, Terry B, Zhang S, Wang C, Fan L, Dicker I, Gali V, Higley H, Parkin N, Tenney D, Krystal M, Colonno R (2008). Entecavir exhibits inhibitory activity against human immunodeficiency virus under conditions of reduced viral challenge. Antimicrob Agents Chemother.

[107] Zhu M, Bifano M, Xu X, Wang Y, LaCreta F, Grasela D, Pfister M (2008). Lack of an effect of human immunodeficiency virus coinfection on the pharmacokinetics of entecavir in hepatitis B virus-infected patients. Antimicrob Agents Chemother.

[108] Keam SJ (2007). Telbivudine. Drugs.

[109] Nash K (2009). Telbivudine in the treatment of chronic hepatitis B. Adv Ther.

[110] Ruiz-Sancho A, Sheldon J, Soriano V (2007). Telbivudine: a new option for the treatment of chronic hepatitis B. Exp Opin Biol Ther.

[111] Bryant ML, Bridges EG, Placidi L, Faraj A, Loi AG, Pierra C, Dukhan D, Gosselin G, Imbach JL, Hernandez B, Juodawlkis A, Tennant B, Korba B, Cote P, Marion P, Cretton-Scott E, Schinazi RF, Sommadossi JP (2001). Antiviral L-nucleosides specific for hepatitis B virus infection. Antimicrob Agents Chemother.

[112] Hernandez-Santiago B, Placidi L, Cretton-Scott E, Faraj A, Bridges EG, Bryant ML, Rodriguez-Orengo J, Imbach JL, Gosselin G, Pierra C, Dukhan D, Sommadossi JP (2002). Pharmacology of beta-L-thymidine and beta-L-2′-deoxycytidine in HepG2 cells and primary human hepatocytes: relevance to chemotherapeutic efficacy against hepatitis B virus. Antimicrob Agents Chemother.

[113] Kim JW, Park SH, Louie SG (2006). Telbivudine: A novel nucleoside analog for chronic hepatitis B. Ann Pharmacother.

[114] Lai CL, Lim SG, Brown NA, Zhou XJ, Lloyd DM, Lee YM, Yuen MF, Chao GC, Myers MW (2004). A dose-finding study of once-daily oral telbivudine in HBeAg-positive patients with chronic hepatitis B virus infection. Hepatology.

[115] Lai CL, Leung N, Teo EK, Tong M, Wong F, Hann HW, Han S, Poynard T, Myers M, Chao G, Lloyd D, Brown NA (2005). A 1-year trial of telbivudine, lamivudine, and the combination in patients with hepatitis B e antigen-positive chronic hepatitis B. Gastroenterology.

[116] Chan HL, Heathcote EJ, Marcellin P, Lai CL, Cho M, Moon YM, Chao YC, Myers RP, Minuk GY, Jeffers L, Sievert W, Bzowej N, Harb G, Kaiser R, Qiao XJ, Brown NA (2007). Treatment of hepatitis B e antigen positive chronic hepatitis with telbivudine or adefovir: a randomized trial. Ann Intern Med.

[117] Lai CL, Gane E, Liaw YF, Hsu CW, Thongsawat S, Wang Y, Chen Y, Heathcote EJ, Rasenack J, Bzowej N, Naoumov NV, Di Bisceglie AM, Zeuzem S, Moon YM, Goodman Z, Chao G, Constance BF, Brown NA (2007). Telbivudine *versus* lamivudine in patients with chronic hepatitis B. N Engl J Med.

[118] Hou J, Yin YK, Xu D, Tan D, Niu J, Zhou X, Wang Y, Zhu L, He Y, Ren H, Wan M, Chen C, Wu S, Chen Y, Xu J, Wang Q, Wei L, Chao G, Constance BF, Harb G, Brown NA, Jia J (2008). Telbivudine *versus* lamivudine in Chinese patients with chronic hepatitis B: Results at 1 year of a randomized, double-blind trial. Hepatology.

[119] Zeuzem S, Gane E, Liaw YF, Lim SG, DiBisceglie A, Buti M, Chutaputti A, Rasenack J, Hou J, O’Brien C, Nguyen TT, Jia J, Poynard T, Belanger B, Bao W, Naoumov NV (2009). Baseline characteristics and early on-treatment response predict the outcomes of 2 years of telbivudine treatment of chronic hepatitis B. J Hepatol.

[120] Liaw YF, Gane E, Leung N, Zeuzem S, Wang Y, Lai CL, Heathcote EJ, Manns M, Bzowej N, Niu J, Han SH, Hwang SG, Cakaloglu Y, Tong MJ, Papatheodoridis G, Chen Y, Brown NA, Albanis E, Galil K, Naoumov NV (2009). 2-Year GLOBE trial results: telbivudine Is superior to lamivudine in patients with chronic hepatitis B. Gastroenterology.

[121] Zhou XJ, Fielman BA, Lloyd DM, Chao GC, Brown NA (2006). Pharmacokinetics of telbivudine in healthy subjects and absence of drug interaction with lamivudine or adefovir dipivoxil. Antimicrob Agents Chemother.

[122] Seifer M, Patty A, Serra I, Li B, Standring DN (2009). Telbivudine, a nucleoside analog inhibitor of HBV polymerase, has a different *in vitro* cross-resistance profile than the nucleotide analog inhibitors adefovir and tenofovir. Antiviral Res.

[123] Thomas H, Foster G, Platis D (2003). Mechanisms of action of interferon and nucleoside analogues. J Hepatol.

[124] Lok ASF, McMahon BJ (2007). Chronic hepatitis B. Hepatology.

[125] Lau GK, Piratvisuth T, Luo KX, Marcellin P, Thongsawat S, Cooksley G, Gane E, Fried MW, Chow WC, Paik SW, Chang WY, Berg T, Flisiak R, McCloud P, Pluck N (2005). Peginterferon Alfa-2a, lamivudine, and the combination for HBeAg-positive chronic hepatitis B. N Engl J Med.

[126] Brunetto MR, Moriconi F, Bonino F, Lau GK, Farci P, Yurdaydin C, Piratvisuth T, Luo K, Wang Y, Hadziyannis S, Wolf E, McCloud P, Batrla R, Marcellin P (2009). Hepatitis B virus surface antigen levels: a guide to sustained response to peginterferon alfa-2a in HBeAg-negative chronic hepatitis B. Hepatology.

[127] Janssen HL, van Zonneveld M, Senturk H, Zeuzem S, Akarca US, Cakaloglu Y, Simon C, So TM, Gerken G, de Man RA, Niesters HG, Zondervan P, Hansen B, Schalm SW (2005). Pegylated interferon alpha-2b alone or in combination with lamivudine for HBeAg-positive chronic hepatitis B: a randomised trial. Lancet.

[128] Liu CJ, Lai MY, Chao YC, Liao LY, Yang SS, Hsiao TJ, Hsieh TY, Lin CL, Hu JT, Chen CL, Chen PJ, Kao JH, Chen DS (2006). Interferon alpha-2b with and without ribavirin in the treatment of hepatitis B e antigen-positive chronic hepatitis B: a randomized study. Hepatology.

[129] Wursthorn K, Lutgehetmann M, Dandri M, Volz T, Buggisch P, Zollner B, Longerich T, Schirmacher P, Metzler F, Zankel M, Fischer C, Currie G, Brosgart C, Petersen J (2006). Peginterferon alpha-2b plus adefovir induce strong cccDNA decline and HBsAg reduction in patients with chronic hepatitis B. Hepatology.

[130] Buster EH, Hansen BE, Buti M, Delwaide J, Niederau C, Michielsen PP, Flisiak R, Zondervan PE, Schalm SW, Janssen HL (2007). Peginterferon alpha-2b is safe and effective in HBeAg-positive chronic hepatitis B patients with advanced fibrosis. Hepatology.

[131] Benhamou Y, Fleury H, Trimoulet P, Pellegrin I, Urbinelli R, Katlama C, Rozenbaum W, Le Teuff G, Trylesinski A, Piketty C (2006). Anti-hepatitis B virus efficacy of tenofovir disoproxil fumarate in HIV-infected patients. Hepatology.

[132] Zhu; Y, Curtis M, Qi X, Miller MD, Borroto-Esoda K (2009). Anti-hepatitis B virus activity *in vitro* of combinations of tenofovir with nucleoside/nucleotide analogues. Antiviral Chem Chemother.

[133] Delaney WE, Ray AS, Yang H, Qi X, Xiong S, Zhu Y, Miller MD (2006). Intracellular metabolism and *in vitro* activity of tenofovir against hepatitis B virus. Antimicrob Agents Chemother.

[134] Birkus G, Hajek M, Kramata P, Votruba I, Holý A, Otova B (2002). Tenofovir diphosphate is a poor substrate and a weak inhibitor of rat DNA polymerases alpha, delta, and epsilon*. Antimicrob Agents Chemother.

[135] Delaney WE, Borroto-Esoda K (2008). Therapy of chronic hepatitis B: trends and developments. Curr Opin Pharmacol.

[136] Marcellin P, Heathcote EJ, Buti M, Gane E, de Man RA, Krastev Z, Germanidis G, Lee SS, Flisiak R, Kaita K, Manns M, Kotzev I, Tchernev K, Buggisch P, Weilert F, Kurdas OO, Shiffman ML, Trinh H, Washington MK, Sorbel J, Anderson J, Snow-Lampart A, Mondou E, Quinn J, Rousseau F (2008). Tenofovir disoproxil fumarate *versus* adefovir dipivoxil for chronic hepatitis B. N Engl J Med.

[137] Lacombe K, Gozlan J, Boyd A, Boelle PY, Bonnard P, Molina JM, Miailhes P, Lascoux-Combe C, Serfaty L, Zoulim F, Girard PM (2008). Comparison of the antiviral activity of adefovir and tenofovir on hepatitis B virus in HIV-HBV-coinfected patients. Antiviral Ther.

[138] Del Poggio P, Zaccanelli M, Oggionni M, Colombo S, Jamoletti C, Puhalo V (2007). Low-dose tenofovir is more potent than adefovir and is effective in controlling HBV viremia in chronic HBeAg-negative hepatitis B. World J Gastroenterol.

[139] Tan J, Degertekin B, Wong SN, Husain M, Oberhelman K, Lok AS (2008). Tenofovir monotherapy is effective in hepatitis B patients with antiviral treatment failure to adefovir in the absence of adefovir-resistant mutations. J Hepatol.

[140] Leemans WF, Janssen HL, Niesters HG, de Man RA (2008). Switching patients with lamivudine resistant chronic hepatitis B virus from tenofovir to adefovir results in less potent HBV-DNA suppression. J Viral Hepat.

[141] Gutiérrez S, Guillemi S, Jahnke N, Montessori V, Harrigan PR, Montaner JS (2008). Tenofovir-based rescue therapy for advanced liver disease in 6 patients coinfected with HIV and hepatitis B virus and receiving lamivudine. Clin Infect Dis.

[142] Menne S, Cote PJ, Korba BE, Butler SD, George AL, Tochkov IA, Delaney WE, Xiong S, Gerin JL, Tennant BC (2005). Antiviral effect of oral administration of tenofovir disoproxil fumarate in woodchucks with chronic woodchuck hepatitis virus infection. Antimicrob Agents Chemother.

[143] Sheldon J, Camino N, Rodés B, Bartholomeusz A, Kuiper M, Tacke F, Núñez M, Mauss S, Lutz T, Klausen G, Locarnini S, Soriano V (2005). Selection of hepatitis B virus polymerase mutations in HIV-coinfected patients treated with tenofovir. Antiviral Ther.

[144] Amini-Bavil-Olyaee S, Herbers U, Sheldon J, Luedde T, Trautwein C, Tacke F (2009). The rtA194T polymerase mutation impacts viral replication and susceptibility to tenofovir in hepatitis B e antigen-positive and hepatitis B e antigen-negative hepatitis B virus strains. Hepatology.

[145] van Bömmel F, Zöllner B, Sarrazin C, Spengler U, Hüppe D, Möller B, Feucht HH, Wiedenmann B, Berg T (2006). Tenofovir for patients with lamivudine-resistant hepatitis B virus (HBV) infection and high HBV DNA level during adefovir therapy. Hepatology.

[146] Korba BE, Schinazi RF, Cote P, Tennant BC, Gerin JL (2000). Effect of oral administration of emtricitabine on woodchuck hepatitis virus replication in chronically infected woodchucks. Antimicrob Agents Chemother.

[147] Gish RG, Trinh H, Leung N, Chan FK, Fried MW, Wright TL, Wang C, Anderson J, Mondou E, Snow A, Sorbel J, Rousseau F, Corey L (2005). Safety and antiviral activity of emtricitabine (FTC) for the treatment of chronic hepatitis B infection: a two-year study. J Hepatol.

[148] Lim SG, Krastev Z, Ng TM, Mechkov G, Kotzev IA, Chan S, Mondou E, Snow A, Sorbel J, Rousseau F (2006). Randomized, double-blind study of emtricitabine (FTC) plus clevudine *versus* FTC alone in treatment of chronic hepatitis B. Antimicrob Agents Chemother.

[149] Marcellin P, Mommeja-Marin H, Sacks SL, Lau GK, Sereni D, Bronowicki JP, Conway B, Trepo C, Blum MR, Yoo BC, Mondou E, Sorbel J, Snow A, Rousseau F, Lee HS (2004). A phase II dose-escalating trial of clevudine in patients with chronic hepatitis B. Hepatology.

[150] Goulis I, Dalekos GN (2008). Entecavir monotherapy for lamivudine-refractory chronic hepatitis B. Expert Rev Anti Infect Ther.

[151] Yoo BC, Kim JH, Chung YH, Lee KS, Paik SW, Ryu SH, Han BH, Han JY, Byun KS, Cho M, Lee HJ, Kim TH, Cho SH, Park JW, Um SH, Hwang SG, Kim YS, Lee YJ, Chon CY, Kim BI, Lee YS, Yang JM, Kim HC, Hwang JS, Choi SK, Kweon YO, Jeong SH, Lee MS, Choi JY, Kim DG, Kim YS, Lee HY, Yoo K, Yoo HW, Lee HS (2007). Twenty-four-week clevudine therapy showed potent and sustained antiviral activity in HBeAg-positive chronic hepatitis B. Hepatology.

[152] Yoo BC, Kim JH, Kim TH, Koh KC, Um SH, Kim YS, Lee KS, Han BH, Chon CY, Han JY, Ryu SH, Kim HC, Byun KS, Hwang SG, Kim BI, Cho M, Yoo K, Lee HJ, Hwang JS, Kim YS, Lee YS, Choi SK, Lee YJ, Yang JM, Park JW, Lee MS, Kim DG, Chung YH, Cho SH, Choi JY, Kweon YO, Lee HY, Jeong SH, Yoo HW, Lee HS (2007). Clevudine is highly efficacious in hepatitis B e antigen-negative chronic hepatitis B with durable off-therapy viral suppression. Hepatology.

[153] Kim BK, Oh J, Kwon SY, Choe WH, Ko SY, Rhee KH, Seo TH, Lim SD, Lee CH (2009). Clevudine myopathy in patients with chronic hepatitis B. J Hepatol.

[154] Seok JI, Lee DK, Lee CH, Park MS, Kim SY, Kim HS, Jo HY, Lee CH, Kim DS (2009). Long-term therapy with clevudine for chronic hepatitis B can be associated with myopathy characterized by depletion of mitochondrial DNA. Hepatology.

[155] European Association for the Study of the Liver (2009). EASL Clinical practice guidelines: management of chronic hepatitis B. J Hepatol.

[156] Lok ASF, McMahon BJ (2009). AASLD Practice guidelines. Chronic hepatitis B: update 2009. Hepatology.

[157] Liaw Y-F, Suh DJ, Omata M (2008). APASL guidelines for HBV management.

[158] Piratvisuth T (2008). Reviews for APASL guidelines: immunomodulatory therapy of chronic hepatitis B. Hepatol Int.

[159] Zoulim F, Perrillo R (2008). Hepatitis B reflections on the current approach to antiviral therapy. J Hepatol.

[160] Lampertico P, Vigano M, Manenti E, Iavarone M, Colombo M (2008). Add-on adefovir prevents the emergence of adefovir resistance in lamivudine-resistant patients: a 4-year study. J Hepatol.

[161] Fournier C, Zoulim F (2007). Antiviral therapy of chronic hepatitis B prevention of drug resistance. Clin Liver Dis.

[162] Papatheodoridis GV, Manolakopoulos S, Dusheiko G, Archimandritis AJ (2008). Therapeutic strategies in the management of patients with chronic hepatitis B virus infection. Lancet Infect Dis.

[163] Lampertico P, Colombo M (2009). HBeAg-negative chronic hepatitis B: why do I treat my patients with nucleos(t)ide analogues. Liver Int.

[164] Liaw YF (2009). On-treatment outcome prediction and adjustment during chronic hepatitis B therapy: now and future. Antiviral Ther.

